# A Perspective on “Hypoxia Resistance is an Inherent Phenotype of the Mouse Flexor Digitorum Brevis Skeletal Muscle”

**DOI:** 10.1093/function/zqad024

**Published:** 2023-05-16

**Authors:** Camila Padilha, Ashleigh M Philp

**Affiliations:** Biology of Ageing Laboratory, Centenary Institute, Royal Prince Alfred Hospital, Missenden Rd, NSW 2050, Australia; Centre for Healthy Ageing, Centenary Institute, Missenden Road, Sydney, NSW 2050, Australia; Faculty of Medicine and Health, Charles Perkins Centre, University of Sydney, Sydney, NSW 2006, Australia; Faculty of Health, School of Sport, Exercise and Rehabilitation Sciences, University of Technology Sydney, Ultimo, NSW 2021, Australia; Faculty Medicine and Health, St Vincent’s Clinical School, University of New South Wales, Sydney, NSW 2052, Australia; Centre for Inflammation, Centenary Institute and University of Technology Sydney, Sydney, NSW 2042, Australia

**Keywords:** perspective, Hypoxias, resistance, inherent, phenotype, mouse

Skeletal muscle is reliant on a constant oxygen supply for movement, cellular respiration, and thermogenesis. Heterogeneous fibre types exist in skeletal muscle as a continuum from slow- to fast-twitch to facilitate specialized function. Type I (oxidative fibres) present a slow-twitch phenotype, characterized by high oxygen capacity and increased fatigue resistance. In contrast, type IIa (fast oxidative glycolytic phenotype) and type IIx (fast glycolytic) present faster twitch speeds and contractile force a faster time to fatigue and a recued reliance on oxidative energy metabolism.^1^ This reliance on oxygen for functional capacity means that a sustained lack of oxygen supply (hypoxia and/or anoxia) to muscle has been linked to numerous pathological conditions such as chronic inflammation, slow tissue repair, microvascular complications, and irreversible damage (ie, muscle necrosis).^2^

It has been proposed that the location of the muscle (proximal or distal) to the site of hypoxic insult might dictate the functional and necrosis response to reduced oxygen availability. To test this hypothesis, a recent study published by Amorese *et al*. in *Function^3^* used femoral artery transection of the lower limb in (10–12 wk old, C57BL/6NJ) mice to induce hypoxia in vivo and anoxia ex vivo to challenge different muscles [eg, extensor digitorum longus (EDL), soleus (SOL), and flexor digitorium brevis (FDB)] to hypoxia and anoxia insults. Following this process, the authors tested the functional consequence of oxygen availability by measuring muscle force production and metabolic changes during anoxia exposure (3 h). After a hypoxic insult, the authors observed higher fibrotic damage in EDL and SOL compared with FDB skeletal muscle, suggesting that muscle location might alter functional capacity in the absence of oxygen.

## FDB Skeletal Muscle Shows Unique Phenotypical Responses to Altered Oxygen Availability

Unlike other skeletal muscles located in the distal region, the FDB skeletal muscle presented preserved contractile function during anoxic conditions compared with EDL and SOL suggesting that this unique phenotype might be attributed at least in part by oxygen tension or energetic supply (glycolysis). To test this directly, Amorese *et al*.^3^ showed that during anoxia insult in the presence of potassium cyanide (KCN), an inhibitor of mitochondrial respiration, the FDB skeletal muscle maintained force production compared to the EDL, despite lower mitochondrial content, respiration and membrane potential. Thus, the authors elegantly demonstrated that the FDB response to oxygen availability differed to other muscles in the lower leg, a response that did not seem to rely on altered mitochondrial function.

## Why Does the FDB Display Altered Adaptation to Reduced Oxygen Availability?

Given the KCN results, the authors speculated that the attenuated force and energy production of the FDB may be attributed to an upregulation of glycolytic metabolism. To test this hypothesis, the authors incubated FDB muscle with the glycolysis inhibitor bromopyruvate (BrP) for 3 h. All three skeletal muscles (FDB, EDL, and SOL) treated with BrP displayed a force reduction at a similar timepoint, as well as similar glycogen depletion in anoxia exposure, confirming that FDB does not depend on glycolysis more than other muscles. To further delineate the unique phenotype of the FDB skeletal muscle, Amorese and colleagues compared the proteome of the three skeletal muscles (FDB, EDL, and SOL) using tandem mass tag (TMT)-labeled mass spectrometry and analyzed differential expression patterns of over 2000 proteins across skeletal muscles. This approach identified that the number of glycolytic proteins normalized to the total number of identified proteins was significantly lower in the FDB compared to EDL but not SOL.

Considering the deleterious effect of the glycolytic inhibitor on the force production of the FDB skeletal muscle, the authors examined the abundance of the transmembrane glucose transporter protein type 1 (GLUT1), a rate limiting step in glucose metabolism. The results showed that GLUT1 protein content in FDB skeletal muscle was 1.9-fold higher than EDL and 2.1-fold higher than SOL. Additionally, the authors investigated whether GLUT1 plays a decisive role in fatigue resistance in FDB utilizing skeletal muscle-specific GLUT1 knockout mice (GLUT1 KO). The deletion of GLUT1 in the FDB skeletal muscle resulted in a marked loss of force production following anoxia exposure whereas this response was preserved in wild-type mice. Furthermore, force production in FDB skeletal muscle was significantly different between genotypes after 100 min of anoxia exposure whereas no genotype effect was found under oxygenated conditions.

## What can the FDB Teach us About Muscle Adaptation to Environmental Stress?

The work from Amorese and colleagues is a wonderful example of how skeletal muscle has evolved to deal with specific environmental stresses to meet energetic demands. This remarkable plasticity to adapt to environmental, physiological, and pathological conditions exhibited by skeletal muscle is conferred by changes in muscle contractile phenotype and metabolic shift.^4^ Protecting skeletal muscle from hypoxia is a critical need to improve tissue survival during states of acute hypoxia or during muscle wasting. Thus, understanding the potential mechanisms by which FDB can maintain function in the absence of oxygen may provide important new information as to how and why hypoxia and anoxia are detrimental to muscle function in clinical scenarios.^5^

Disuse atrophy, characterized as a loss of muscle mass, reduced muscle strength, and metabolic dysfunction,^6^ are common features of sedentary behavior resulting from hospitalization, limb immobilization, or bedrest. Systemic hypoxia, has been identified as contributing to poor musculoskeletal states in multiple clinical populations, including chronic obstructive pulmonary disease (COPD)^7^ and heart failure.^8^ Amorese and colleagues have identified a unique phenotype of the FDB muscle and its ability to maintain its function in the complete absence of oxygen. Thus, studies such as this, where different muscles are examined in response to fundamental metabolic/environmental stressors may identify novel biology to explain why muscles can or cannot adapt to alterations in demand. Certainly, these results support the inclusion of numerous tissue analysis in rodent metabolic studies factoring in the relevance of anatomical properties of muscle in specific disease pathologies. To support this notion, recent work from Naruse *et al*. (2023) have reported that muscle-specific atrophy is varied in human skeletal muscle in response to aging.^9^ Furthermore, the trajectory of muscle loss was variable when comparing across muscle groups (quadriceps, 0.66%/yr; hamstrings 1.18%/yr), providing useful information for development of strategies to prevent muscle loss. Indeed, measuring mechanistic responses in both atrophy-prone and atrophy-resistant muscles, has recently been proposed as a valuable approach to identifying novel pathways in disuse atrophy models, which may provide novel targets for therapeutic intervention.^10^ Thus, rather than considering divergent responses as a biological anomaly, perhaps studying muscle such as the FDB can provide valuable information into the unique adaptive potential of skeletal muscle in health and disease.
